# Prevalence and Risk Factors for Obstructive Sleep Apnea in São Paulo: Findings From the 4th Edition of the EPISONO Study

**DOI:** 10.1111/jsr.70255

**Published:** 2026-01-27

**Authors:** Sergio Tufik, Allan Saj Porcacchia, Gabriel Natan Pires, Monica Levy Andersen

**Affiliations:** ^1^ Departamento de Psicobiologia Universidade Federal de São Paulo (UNIFESP) São Paulo Brazil; ^2^ Instituto do Sono Associação Fundo de Incentivo à Pesquisa (AFIP) São Paulo Brazil

**Keywords:** epidemiology, obstructive sleep apnea, prevalence, public health, sleep

## Abstract

Obstructive sleep apnea (OSA) affects around 936 million individuals worldwide, making it the most prevalent breathing‐related sleep disorder. The aim was to estimate the prevalence of OSA in São Paulo, Brazil, based on data from the São Paulo Epidemiologic Sleep Study (EPISONO) 4th edition. This was a cross‐sectional study with a probabilistic sample obtained through a 4‐stage cluster sampling method aiming to represent the population according to age (20–80 years), gender and socio‐economic condition. Participants underwent in‐lab full‐night polysomnography and completed sleep‐related questionnaires. The diagnosis of OSA followed the most recent guidelines of the American Academy of Sleep Medicine. Prevalences were calculated and a multivariable logistic regression evaluated risk factors associated with OSA. The total sample included 769 individuals (452 women). The general adjusted prevalence of OSA was 37.12%, being higher in men (44.88%) than women (30.79%). Greater prevalence was observed in advanced age groups. Moderate and severe OSA affected 11.5% and 7.9% of the participants, respectively. Ageing, body mass index and being male were associated with a higher risk of OSA, especially in moderate‐to‐severe cases. The 4th edition of EPISONO found that more than one‐third of the population had OSA, reaching 45% in males. These results underscore the need for public health actions based on scalable prevention strategies and equitable access to OSA therapies.

## 1 Introduction

The 3rd edition of the São Paulo Epidemiologic Sleep Study (EPISONO) was undertaken in the first decade of the 21st century. This landmark investigation marked the first large‐scale study in the Americas to use polysomnographic data to evaluate sleep patterns in a representative sample of a highly urbanised population (Tufik et al. 2010). The findings revealed an overall prevalence of obstructive sleep apnea (OSA) of 32.9% in the sampled population, with prevalence rates escalating to 40.6% among male participants (Tufik et al. 2010). Globally, recent estimates indicate that approximately 936 million individuals meet the diagnostic criteria for OSA, defined by an apnea‐hypopnea index (AHI) ≥ 5, underscoring its pervasive burden (Benjafield et al. 2019; Berry et al. 2012). China, the United States, Brazil and India are ranked as the countries with the highest number of individuals with OSA (Benjafield et al. 2019).

While OSA, marked by intermittent obstructions in the upper airway during sleep, may manifest asymptomatically, it is strongly associated with multisystem morbidity, including cardiovascular and metabolic disorders, neurocognitive impairments and occupational hazards (Castro, Castro, et al. 2013; Castro, Poyares, et al. 2013; Cintra et al. 2012; de Mello et al. 2012; Malhotra et al. 2015; Togeiro et al. 2013; Ahmed et al. 2025). OSA, alongside insomnia, is one of the most prevalent sleep disorders and has substantial public health implications (Tufik et al. 2010; Castro, Castro, et al. 2013; Castro, Poyares, et al. 2013; Faria et al. 2021). The key risk factors for OSA are advanced age, overweight/obesity and male sex (Lévy et al. 2015; Messineo et al. 2024). The first two of these factors have been subject to important changes in the last decades, due to a demographic shift towards ageing populations and a global rise in overweight and obesity across all age cohorts (Sander et al. 2015; GBD 2015 Obesity Collaborators 2017). These epidemiological trends make it necessary to continuously monitor the prevalence of OSA.

The EPISONO project remains a cornerstone of sleep epidemiology, employing updated methods and advancing the understanding of sleep health and its related pathologies, especially OSA, in a representative sample population. The aim of this study is to provide an update on the prevalence of OSA in São Paulo, based on data from the 4th edition of EPISONO (2018–2019).

## 2 Methods

### 2.1 Population and Sampling Procedures

This single‐centre study was approved by the Research Ethics Committee of the Federal University of São Paulo (project n. 0985/2017, certificate 73692317.5.0000.5505). Data collection was performed between May 2018 and April 2019. All participants read and signed an informed consent form. The methods used in the 4th edition replicate those of the 3rd edition and have been described in detail elsewhere (Santos‐Silva et al. 2009). The information herein presented refers only to the procedures of interest in respect to the current study.

The sampling procedure for the 4th edition of EPISONO followed the same protocol as the previous edition (Tufik et al. 2010; Santos‐Silva et al. 2009), using a probabilistic technique and 4‐stage stratification (districts, blocks, households and individuals). The goal was to obtain a representative sample of the city's population regarding age, sex and socioeconomic status. Twelve municipal districts were randomly selected from each of the four homogeneous economically active population regions (based on the São Paulo social vulnerability index). From each of these districts, two census blocks were sampled and 13 households per block were selected. Only permanently occupied, privately owned residences were included, excluding institutions, such as hospitals, schools and hotels. High‐risk or hazardous areas were excluded to ensure interviewer safety. Household selection involved random sampling of the 1st unit, followed by a predetermined interval calculated by dividing the total households in the block by 25. In apartment buildings, each unit was treated as a distinct residence, with counting conducted from the top floor downwards. Residents were randomly selected for interviews. For that, all eligible residents in a given household were sorted from the youngest to the oldest and the elected resident was selected based on previously prepared randomisation tables. A replacement protocol was implemented for cases involving 3 failed contact attempts, refusal to participate, physical/mental impairment, family interference, or when there were travel, hospitalisation or scheduling conflicts. In such instances, a substitute was selected from the nearest neighbouring household using the same randomisation criteria.

A sample weight for sample expansion was calculated for each included individual. It was calculated in two steps. First, the direct sampling weight was calculated based on the proportional representation of each individual according to 4 criteria: (1) The number of selected districts among all districts available for each social vulnerability strata; (2) The number of households in a given block among all available households in the district containing that block; (3) The number of selected households in a given block among all households in the same block; (4) The number of selected participants per household (*n* = 1) among eligible participants in the same household. Following this, the direct weight for each household was calibrated according to the estimated populational distribution according to sex, age and social vulnerability status in the year of 2017, resulting in the final weight used for the analyses.

### 2.2 Inclusion and Exclusion Criteria

**FIGURE 1:**
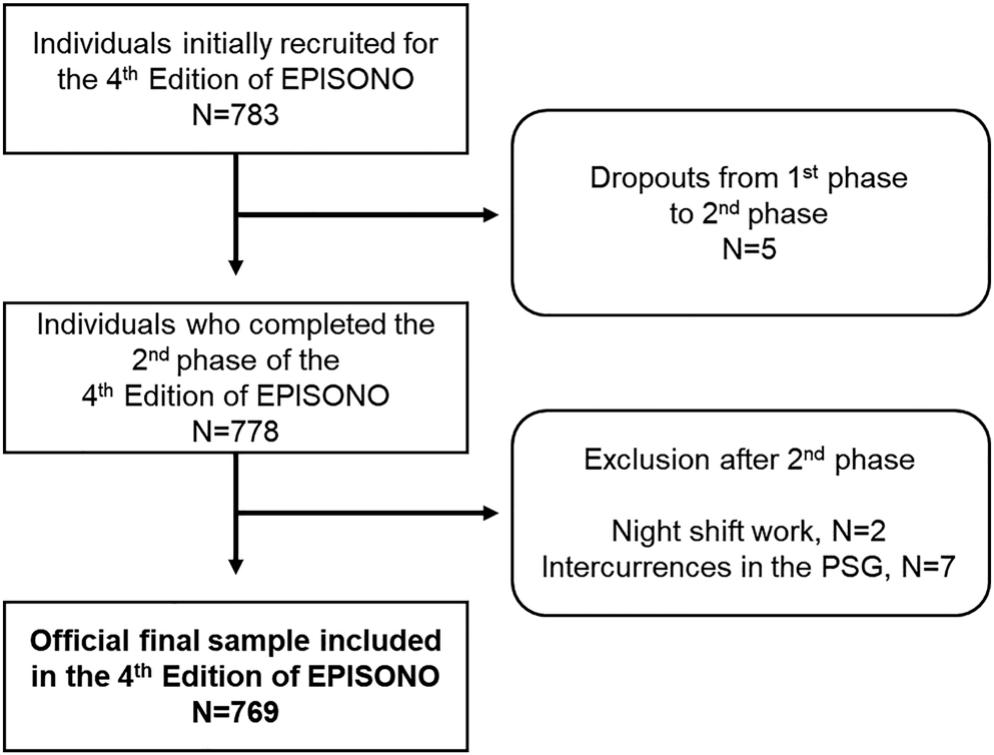
Flowchart of the sample included in the study. Initially, 783 individuals were recruited to participate in the 4th Edition of EPISONO, but the official final sample included 769 participants with validated data. EPISONO, epidemiological sleep study; PSG, polysomnography.

Eligible participants were residents of São Paulo aged 20–80 years. The exclusion criteria were: pregnant or breastfeeding women (up to 6 months postpartum), individuals with physical disabilities that prevented the performance of a polysomnography (PSG) in the sleep laboratory, individuals with intellectual disabilities that would prevent them from understanding research instructions, fixed night shift workers and employees (rather than residents) in the selected households (Figure 1).

### 2.3 Data Collection Methods

The 1st phase of data collection started with trained interviewers from an outsourced pooling company (Datafolha) visiting participants in the selected households. After obtaining consent from the selected participant, demographic and socioeconomic data based on the Brazilian Economic Classification Criteria (a socioeconomic stratification system widely used in Brazil) were collected, and the following questionnaires were administered: the Pittsburgh Sleep Quality Index (PSQI), the Epworth Sleepiness Scale (ESS), the Insomnia Severity Index (ISI), the Berlin Questionnaire, the Chalder Fatigue Scale (CFS) and the Universidade Federal de São Paulo (UNIFESP) Sleep Questionnaire. In the 2nd phase, participants underwent full‐night type I PSG at the Instituto do Sono (São Paulo, Brazil) and completed additional questionnaires. Anthropometric measurements (height and weight) were collected to calculate body mass index (BMI) following the World Health Organisation guidelines (Obesity: Preventing and Managing the Global Epidemic. Report of a WHO Consultation 2000). Blood samples for biochemical analysis were collected the morning after PSG.

### 2.4 Polysomnography

Overnight PSG was conducted using the EMBLA digital system (N7000, Embla Systems Inc., Broomfield, USA), adhering to current guidelines of the American Academy of Sleep Medicine (AASM) (Berry et al. 2020). Sensors were non‐invasively attached with adhesive tape or elastic collodion. The following physiological variables were continuously monitored: six electroencephalogram channels (F3‐A2, F4‐A1, C3‐A2, C4‐A1, O1‐A2, O2‐A1), three channels of chin electromyography (EMG), bilateral EMG channels of masseter muscles, bilateral EMG channels of anterior tibialis muscle, bilateral EMG channels of the flexor digitorum superficialis muscles and one electrocardiogram channel (modified D2 lead). Nasal airflow was detected via two channels using EMBLA thermistors and pressure transducers. Thoracoabdominal respiratory effort was recorded with two EMBLA X‐trace belts around the chest and abdomen. Two additional channels were allocated to snoring sensors and body position monitors (EMBLA). Oxygen saturation (SaO_2_) was measured using an EMBLA pulse oximeter. Sleep staging was visually performed by four AASM‐certified technicians following the 2012 AASM manual. Apneas and hypopneas were scored according to 2012 AASM criteria (Berry et al. 2012, 2020).

### 2.5 OSA Diagnosis

The diagnosis of OSA was performed in concordance with the methods used in EPISONO 3rd edition (Tufik et al. 2010), in order to assure comparability between editions. Subjects with an AHI between 15 and 29.9 were diagnosed with moderate OSA, and those with an AHI ≥ 30 with severe OSA. Individuals with an AHI between 5 and 14.9 were diagnosed with mild OSA if they presented symptoms or complaints compatible with at least one of the following characteristics: loud snoring, excessive daytime sleepiness, fatigue, or experienced breathing interruptions during sleep. Individuals with an AHI < 5 regardless of any concurrent symptoms or complaints, or individuals with an AHI < 15 with none of the listed symptoms or complaints, were considered as not having OSA. Loud snoring was classified as positive when participants responded that their snoring was either ‘louder than talking’ or ‘very loud—can be heard in adjacent rooms’ to question 2 of the Berlin Questionnaire (Netzer et al. 1999). Breathing interruptions were deemed positive for participants who reported having at least one breathing cessation a month in response to question 5 of the Berlin Questionnaire (Netzer et al. 1999). Daytime sleepiness was considered positive for participants with an ESS ≥ 10, or for those who reported having problems staying awake more than once a week in response to question 8 of the PSQI (as in EPISONO 3rd edition). Fatigue was considered positive for participants who scored > 4 in the CFS (Chalder et al. 1993).

### 2.6 Statistical Analysis

OSA prevalence was estimated as the ratio of confirmed cases to the total sample, considering both the absolute counts of positive OSA diagnosis and the estimated populational prevalence incorporating sampling weights (adjusted prevalence). To assess OSA‐associated factors, two multivariable logistic regression models were developed: one with a binary dependent variable (OSA presence or absence) and another with an ordinal outcome (no OSA, mild, moderate or severe OSA). Independent variables included age, BMI, gender (men or women) and socioeconomic class (A, B, C or D/E). Analyses were performed in Jamovi (v. 2.6.25), with a significance level set at 5%.

## 3 Results

The total sample of the 4th edition of EPISONO comprised 769 individuals (452 women). The mean age of the sample was 48.8 years old (95% CI = [47.75; 49.86]). Information regarding age, BMI, gender and socio‐economic status is presented in Table 1.

**TABLE 1:** Distributions of age, body mass index, gender and socio‐economic data in the sample of the 4th edition of EPISONO (*N* = 769).

	Total sample		Without OSA		Mild OSA		Moderate OSA		Severe OSA	
	Mean [95% CI]	SD	Mean [95% CI]	SD	Mean [95% CI]	SD	Mean [95% CI]	SD	Mean [95% CI]	SD
Age (years)	48.80 [47.75; 49.86]	14.86	45.45 [44.14; 46.76]	14.66	51.2 [48.9; 53.5]	13.02	55.91 [53.00; 58.82]	13.72	58.49 [55.55; 61.43]	12.51
BMI (kg/m^2^)^a^	27.56 [27.14; 27.98]	5.90	27.11 [26.61; 27.62]	5.59	27.11 [26.61; 27.62]	6.24	27.11 [26.61; 27.62]	5.32	27.11 [26.61; 27.62]	7.58

	*N* (%)	*N* (%)	*N* (%)	*N* (%)	*N* (%)
Age (years)					
20‐29	90 (11.70)	77 (15.91)	9 (7.20)	2 (2.27)	2 (2.78)
30‐39	143 (18.60)	111 (22.93)	19 (15.20)	10 (11.36)	3 (4.17)
40‐49	159 (20.68)	104 (21.49)	27 (21.60)	16 (18.18)	12 (16.67)
50‐59	172 (22.37)	98 (20.25)	32 (25.60)	23 (26.14)	19 (26.39)
60‐69	142 (18.47)	67 (13.84)	29 (23.20)	22 (25.00)	24 (33.33)
70‐80	63 (8.19)	27 (5.58)	9 (7.20)	15 (17.05)	12 (16.67)
Gender					
Women	452 (58.78)	317 (65.50)	63 (50.40)	39 (44.32)	33 (45.83)
Men	317 (41.22)	167 (34.50)	62 (49.60)	49 (55.68)	39 (54.17)
BMI (kg/m^2^)^a^					
Normal	259 (34.08)	172 (35.98)	40 (32.26)	25 (28.74)	22 (30.99)
Overweight	288 (37.89)	179 (37.45)	47 (37.90)	35 (40.23)	27 (38.03)
Obese	213 (28.03)	127 (26.57)	37 (29.84)	27 (31.03)	22 (30.99)
SEC					
A	94 (12.22)	54 (11.16)	20 (16.00)	11 (12.5)	9 (12.5)
B	351 (45.64)	232 (47.93)	47 (37.60)	46 (52.27)	26 (36.11)
C	290 (37.71)	176 (36.36)	51 (40.80)	30 (34.09)	33 (45.83)
D/E	34 (4.42)	22 (4.55)	7 (5.60)	1 (1.14)	4 (5.56)

Abbreviations: BMI, body mass index; CI, confidence interval; SD, standard deviation; SEC, socio‐economic classification.

^a^ Especifically for BMI data, *N* = 760.

**FIGURE 2:**
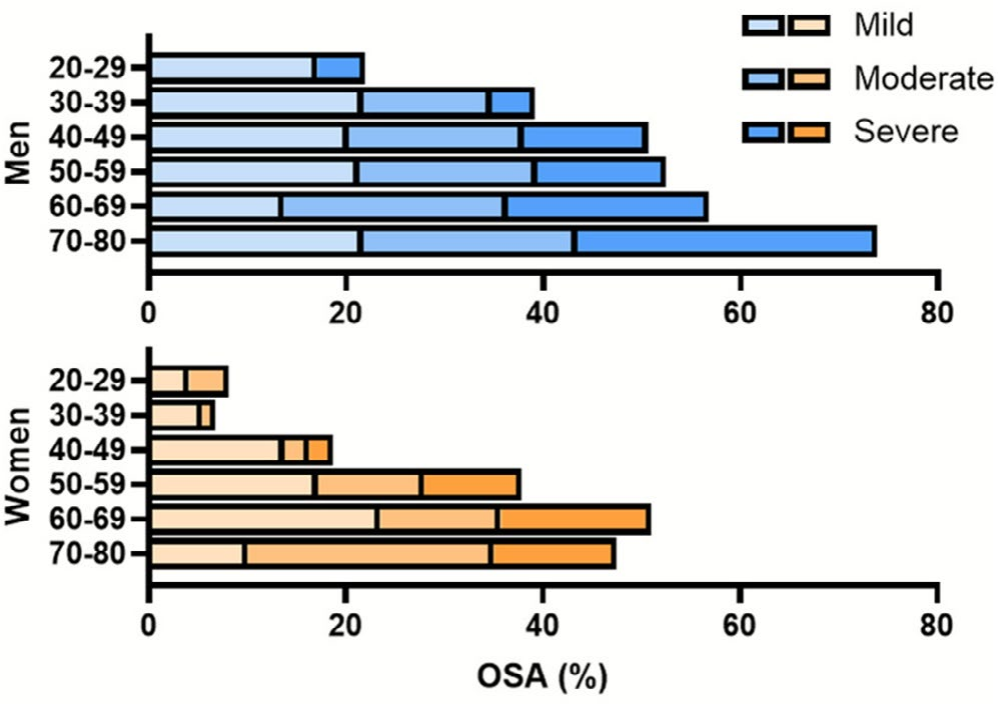
Prevalence of OSA by severity, sex and age (years). Prevalence estimates are presented in blue for men and orange for women. The results in this figure are based on unadjusted prevalence estimates. OSA, obstructive sleep apnea.

Prevalence estimates of OSA in the total sample adjusted by sample weights were 37.12% (95% CI = [33.71; 40.53]). OSA was more common in men (44.88%; 95% CI = [39.38; 50.38]) than in women (30.79%; 95% CI = [26.57; 35.01]). Mild, moderate, and severe OSA were diagnosed in 17.67%, 11.53% and 7.92% of the sample, respectively (Table 2). The prevalence of OSA by age is depicted in Table 3, and by disease severity, sex and age in Figure 2.

**TABLE 2:** Prevalence of OSA in the total sample and by gender.

		Total	Women	Men
Total OSA	Prev.	37.06 [33.65; 40.47]	29.87 [25.65; 34.09]	47.32 [41.82; 52.81]
	Adj Prev.^a^	37.12 [33.71; 40.53]	30.79 [26.57; 35.01]	44.88 [39.38; 50.38]
Mild OSA	Prev.	16.25 [13.65; 18.86]	13.94 [10.75; 17.13]	19.56 [15.19; 23.92]
	Adj Prev.^a^	17.67 [15.06; 20.28]	15.4 [12.2; 18.59]	20.45 [16.09; 24.82]
Moderate OSA	Prev.	11.44 [9.19; 13.69]	8.63 [6.04; 11.22]	15.46 [11.48; 19.44]
	Adj Prev.^a^	11.53 [9.28; 13.78]	8.85 [6.27; 11.44]	14.81 [10.83; 18.79]
Severe OSA	Prev.	9.36 [7.30; 11.42]	7.30 [4.90; 9.70]	12.30 [8.69; 15.92]
	Adj Prev.^a^	7.92 [5.86; 9.98]	6.54 [4.14; 8.94]	9.62 [6.00; 13.24]

*Note:* Values represented as % [95% CI].

Abbreviations: Adj. Prev., adjusted prevalence; OSA, obstructive sleep apnea; Prev., prevalence.

^a^ Prevalences are adjusted with sample weights.

**TABLE 3:** Prevalence of OSA in each age stratum.

Age groups (years)		Total	Women	Men
20–29	Prev.	14.44 [7.18; 21.71]	8.16 [0.50; 15.83]	21.95 [9.28; 34.62]
20–29	Adj Prev.^a^	10.80 [3.54; 18.07]	5.31 [0.00; 12.97]	17.05 [4.38; 29.72]
30–39	Prev.	22.38 [15.55; 29.21]	6.76 [1.04; 12.48]	39.13 [27.61; 50.65]
30–39	Adj Prev.^a^	22.01 [15.18; 28.84]	5.75 [0.03; 11.47]	36.93 [25.42; 48.45]
40–49	Prev.	34.59 [27.20; 41.98]	18.75 [10.20; 27.30]	50.63 [39.61; 61.66]
40–49	Adj Prev.^a^	39.44 [32.04; 46.83]	18.58 [10.02; 27.13]	56.26 [45.24; 67.29]
50–59	Prev.	43.02 [35.62; 50.42]	37.84 [28.82; 46.86]	52.46 [39.93; 64.99]
50–59	Adj Prev.^a^	44.70 [37.30; 52.10]	42.85 [33.82; 51.87]	47.63 [35.10; 60.16]
60–69	Prev.	52.82 [44.61; 61.03]	51.02 [41.12; 60.92]	56.82 [42.18; 71.45]
60–69	Adj Prev.^a^	53.38 [45.17; 61.59]	53.41 [43.52; 63.31]	53.31 [38.68; 67.95]
70–80	Prev.	57.14 [44.92; 69.36]	47.50 [32.02; 62.98]	73.91 [55.97; 91.86]
70–80	Adj Prev.^a^	58.41 [46.19; 70.63]	54.97 [39.5; 70.45]	63.67 [45.72; 81.61]

*Note:* Values represented as % [95% CI].

Abbreviations: Adj. Prev., adjusted prevalence; OSA, obstructive sleep apnea; Prev., prevalence.

^a^ Prevalences are adjusted with sample weights.

A multivariable binary logistic regression model was used to assess the contribution of age, BMI, sex and socioeconomic status variables to the risk of individuals in the sample having OSA (Table 4). Except for socioeconomic status, the other variables showed statistical significance. Each additional year of age represented an increased odds ratio (OR) of 1.05 (95% CI = [1.04; 1.06]; *p* < 0.001) for OSA, while each additional 1 kg/m^2^ in BMI was associated with an increased OR of 1.04 for OSA (95% CI = [1.01; 1.07]; *p* < 0.001). Regarding gender, men had almost three times the risk of being diagnosed with OSA of any severity compared to women (OR = 2.99; 95% CI = [2.14; 4.20]; *p* < 0.001).

**TABLE 4:** Results from the multivariable binary logistic regression model for the risk of obstructive sleep apnea in the 4th edition of EPISONO.

	Estimates	SE	OR [95% CI]	*p*
Intercept	−5.130	0.691	0.006 [0.002; 0.023]	< 0.001
Age	0.053	0.006	1.055 [1.042; 1.067]	< 0.001
BMI	0.043	0.014	1.044 [1.015; 1.072]	0.002
Gender				
Men–Women	1.098	0.172	2.999 [2.14; 4.203]	< 0.001
SEC				
A—D/E	0.439	0.461	1.551 [0.628; 3.832]	0.342
B—D/E	0.151	0.421	1.163 [0.510; 2.655]	0.720
C—D/E	0.433	0.424	1.541 [0.671; 3.541]	0.308

Abbreviations: BMI, body mass index; CI, confidence interval; OR, odds ratio; SE, standard error; SEC, socio‐economic classification.

An ordinal logistic regression model was constructed considering the categories of OSA severity as dependent variables, evaluating the contribution of the same independent variables of the previous model (Table 5). Age was statistically significant, and each additional year was associated with a 6% increase in the odds of experiencing greater OSA severity (OR = 1.06; 95% CI = [1.05; 1.07]; *p* < 0.001). BMI was also linked with increased severity of this sleep disorder, with each additional unit corresponding to a 4% rise in the likelihood of presenting with more severe levels of OSA (OR = 1.04; 95% CI = [1.01; 1.07]; *p* = 0.004). Regarding gender, males had 2.96 times higher odds of exhibiting more severe levels of OSA compared to females (95% CI = [2.17; 4.07]; *p* < 0.001). Socio‐economic status did not present statistical significance in the model.

**TABLE 5:** Results of the ordinal logistic regression model for the risk of mild, moderate or severe OSA in the 4th edition of EPISONO.

	Estimates	SE	OR [95% CI]	*p*
Age	0.056	0.006	1.057 [1.046; 1.070]	< 0.001
BMI	0.038	0.013	1.039 [1.012; 1.066]	0.004
Gender				
Men–Women	1.087	0.161	2.964 [2.168; 4.072]	< 0.001
SEC				
A—D/E	0.368	0.442	1.445 [0.618; 3.533]	0.405
B—D/E	0.138	0.407	1.149 [0.529; 2.641]	0.734
C—D/E	0.423	0.409	1.526 [0.700; 3.522]	0.302

Abbreviations: BMI, body mass index; CI, confidence interval; OR, odds ratio; OSA, obstructive sleep apnea; SE, standard error; SEC, socio‐economic classification.

## 4 Discussion

The prevalence rates of OSA documented in the 4th edition of the EPISONO study align with trends identified in prior epidemiological investigations, including earlier editions of EPISONO (Tufik et al. 2010). These rates exhibit a pronounced male predominance and increase proportionally with age in both sexes. Multivariable logistic regression models further confirmed the association between OSA severity and key demographic and anthropometric determinants, namely, older age, higher BMI and male sex, thereby reaffirming their status as established risk factors for this sleep‐related breathing disorder (Benjafield et al. 2019; Malhotra et al. 2024).

This latest edition of EPISONO demonstrated a marked increase in OSA prevalence across the general population and within gender‐specific subgroups compared to the previous edition (Tufik et al. 2010). In the 3rd edition of EPISONO, the prevalence of OSA was estimated at 32.9%, while in the current one it was estimated at 37.1%. Possible explanations for these changes are related to the sample composition and to the hypopnea scoring criteria. The sample for the current edition comprised 49.03% of individuals aged ≥ 50 years and 65.92% with BMI categorised as overweight or obese, both recognised OSA risk factors. These proportions are higher than in the 3rd edition (29.1% for individuals aged ≥ 50 years, and 59.9% for overweight or obesity) (Tufik et al. 2010), which may have contributed to the greater prevalence reported herein. However, the proportion of individuals at older age ranges was weighted by population proportions, thus reflecting current age distribution in the population, rather than oversampling older adults. Regarding hypopnea scoring criteria, results might reflect updated AASM diagnostic criteria for scoring hypopneas (Berry et al. 2012, 2020). At EPISONO 3rd edition, the alternative rule for hypopnea scoring described in the 1st edition of the AASM scoring manual was used (a 10 s 50% drop in nasal pressure signal, associated with either a 3% desaturation or an arousal), while the EPISONO 4th edition used the recommended rule of the 2nd edition of the AASM scoring manual (a 10 s 30% drop in nasal pressure signal, associated with either a 3% desaturation or an arousal). Previous studies have already demonstrated that the hypopnea scoring criteria have a significant impact on OSA prevalence (Benjafield et al. 2019), especially when changes in the desaturation threshold (3% vs. 4%) or the presence of associated events (especially arousal) are regarded. However, in the current case the only difference criteria was the nasal pressure drop threshold (Berry et al. 2012, 2020), which seems to have little influence on the final estimated prevalence. Considering that the possible effect of sampling bias and different hypopnea scoring criteria is limited, it is likely that the changes in the prevalence of OSA between EPISONO 3rd and 4th editions reflect actual changes in demographic characteristics between 2007 and 2018.

Males had three times the odds of having OSA of worse severity compared to females. These sex‐specific risk estimates mirror those reported in the 3rd edition of EPISONO in 2007, underscoring the persistence of this demographic pattern. While Benjafield et al. did not directly assess sex‐based risk differentials, their synthesis of 18 studies on moderate‐to‐severe OSA prevalence demonstrated male predominance in 10 studies, with most reporting at least double the prevalence in males, and only three studies showing parity among sexes (Benjafield et al. 2019). These results reinforce the robust association between male sex and OSA susceptibility, both in the Brazilian population and globally and further validate the reproducibility of these risk dynamics across distinct temporal and geographic contexts. The findings presented here emphasise the empirical validation of the methodological design and statistical frameworks employed in the 4th edition of the EPISONO study, as evidenced by the replication of OSA‐related outcomes and statistically significant associations with established risk factors previously identified in the 2007 study (Tufik et al. 2010).

The demographic shift towards an ageing population has become a prominent reality in most European and North American nations, as well as in several Asian countries, such as China, where median ages approached or exceeded 40 years by 2023 (https://ourworldindata.org/age‐structure). This trend, initially exemplified by Japan in the early 21st century as one of the few nations with a median age surpassing this threshold, is projected to intensify globally, resulting in a growing proportion of older adults (Sander et al. 2015). As a result, age‐related health conditions, including OSA and the prevalence of its associated comorbidities: cardiovascular diseases, metabolic dysregulation and neurocognitive decline, are anticipated to increase, as we have seen herein (Malhotra et al. 2015).

Sleep health, a critical determinant of overall well‐being, is influenced by multiple interrelated factors. Beyond OSA, these include sleep deprivation, social jetlag, chronotype misalignment, occupational schedules and subjective perceptions of sleep quality (Fernandes et al. 2024, 2023). Previous studies conducted with samples from Brazil have corroborated widespread suboptimal sleep quality within the population, attributable to sociobehavioural factors (Fernandes et al. 2023; Drager et al. 2022) and the burden of sleep disorders, especially OSA and insomnia (Lopes et al. 2008; Lucena et al. 2020; Luciano et al. 2024). Although PSG was included in the list of approved procedures within Brazil's Unified Health System (*Sistema Único de Saúde*) in 2021, persistent disparities in the availability of diagnostic infrastructure across regions hinder equitable access to OSA diagnosis and treatment (Drager et al. 2024). These findings underscore the urgent need for policy initiatives to expand healthcare infrastructure and ensure universal access to diagnostic and therapeutic resources for sleep‐related breathing disorders. This is even more relevant considering the great prevalence of OSA (Tufik et al. 2010) and its negative impacts on the cardiovascular system, neurocognitive disorders and on labour‐ or driving‐related accidents, which have a higher risk of occurrence due to the consequences of OSA (Castro, Castro, et al. 2013; Castro, Poyares, et al. 2013; Cintra et al. 2012; de Mello et al. 2012; Malhotra et al. 2015; Togeiro et al. 2013; Ahmed et al. 2025).

This study is strengthened by the sampling strategy and the implementation of full‐night PSG for all participants, which enhanced the methodological rigour and reliability of the findings. The exclusion of high‐risk areas from data collection might increase the likelihood of sampling bias. However, it shall be noticed that lower socioeconomic status (classes D and E) does not conditionally mean living in endangered or high‐risk areas. Even excluding these areas, the sample from lower socioeconomic classes is relevant in size and their contribution to the final results has been weighted by population estimates. Thus, we believe that this factor is unlikely to have substantially influenced the overall validity of the results. These considerations should be considered when interpreting the data. Sustained public health efforts are warranted to address modifiable OSA risk factors, including elevated BMI (Messineo et al. 2024; Malhotra et al. 2024) and smoking (Zeng et al. 2023), which also contribute to cardiovascular morbidity. Educational campaigns aimed at raising awareness of these risks, alongside structural interventions to mitigate sleep health inequities, are essential to curbing the growing burden of OSA and its downstream health consequences in ageing populations.

## 5 Conclusions

The latest edition of the EPISONO study revealed that OSA affected over 1/3 of the total sample population, with prevalence rates escalating to nearly 45% among male participants. Advanced age, elevated BMI and male sex were reaffirmed as significant risk factors for OSA, particularly for moderate‐to‐severe clinical manifestations of the disorder. These findings align with observations from the 3rd edition of EPISONO conducted in 2007, thereby reinforcing the methodological rigour of the study design. The consistency of these outcomes underscores the necessity for public health initiatives focused on sleep health, including the implementation of evidence‐based preventive strategies and the improvement of accessibility to therapeutic interventions for individuals with OSA.

## Author Contributions

**Sergio Tufik:** conceptualisation, resources, methodology, project administration, supervision. **Allan Saj Porcacchia:** conceptualisation, investigation, data curation, formal statistical analysis, writing – original draft, writing – review and editing. **Gabriel Natan Pires:** conceptualisation, methodology, validation, investigation, data curation, formal statistical analysis, writing – original draft, writing – review and editing, project administration. **Monica Levy Andersen:** conceptualisation, methodology, writing – review and editing, project administration, supervision.

## Funding

This work was supported by the Associação Fundo de Incentivo à Pesquisa (AFIP), São Paulo, Brazil. S.T. and M.L.A. are recipients of Conselho Nacional de Desenvolvimento Científico e Tecnológico (CNPq) fellowships. M.L.A. and A.S.P. are recipients of grants from the Fundação de Amparo à Pesquisa do Estado de São Paulo (FAPESP; grants #2020/13467‐8 and #2021/05920‐7, respectively). No sponsorship was received for the publication of this manuscript.

## Conflicts of Interest

G.N.P. was a shareholder at SleepUp (a Brazilian digital CBTi company), but declares that his position was in no way related to the aims, design or conduct of this study. The remaining authors declare no conflicts of interest.

## Supporting information


**Table S1:** Collinearity parameters from the binary logistic regression model.

## Data Availability

The data that support the findings of this study are available from the corresponding author upon reasonable request.

## References

[jsr70255-bib-0024] 2000. “Obesity: Preventing and Managing the Global Epidemic. Report of a WHO Consultation.” World Health Organization Technical Report Series 894: i–xii. 1–253. 11234459

[jsr70255-bib-0001] Ahmed, S. , D. Gozal , and A. Khalyfa . 2025. “Mechanistic Links Between Obstructive Sleep Apnea, Cellular Senescence and Aging: The Role of Cardiometabolic Dysfunction.” Sleep Medicine Reviews 84: 102170. 41032946 10.1016/j.smrv.2025.102170

[jsr70255-bib-0002] Benjafield, A. V. , N. T. Ayas , P. R. Eastwood , et al. 2019. “Estimation of the Global Prevalence and Burden of Obstructive Sleep Apnoea: A Literature‐Based Analysis.” Lancet Respiratory Medicine 7, no. 8: 687–698. 31300334 10.1016/S2213-2600(19)30198-5 PMC7007763

[jsr70255-bib-0003] Berry, R. B. , R. Budhiraja , D. J. Gottlieb , et al. 2012. “Rules for Scoring Respiratory Events in Sleep: Update of the 2007 AASM Manual for the Scoring of Sleep and Associated Events.” Journal of Clinical Sleep Medicine 8, no. 5: 597–619. 23066376 10.5664/jcsm.2172 PMC3459210

[jsr70255-bib-0004] Berry, R. B. , S. F. Quan , A. R. Abreu , M. L. Bibbs , L. DelRosso , and S. M. Harding . 2020. The AASM Manual for the Scoring of Sleep and Associated Events: Rules, Terminology and Technical Specifications, Version 2.6. American Academy of Sleep Medicine. https://aasm.org/clinical‐resources/scoring‐manual/.

[jsr70255-bib-0005] Castro, L. S. , J. Castro , M. Q. Hoexter , et al. 2013. “Depressive Symptoms and Sleep: A Population‐Based Polysomnographic Study.” Psychiatry Research 210, no. 3: 906–912. 24041750 10.1016/j.psychres.2013.08.036

[jsr70255-bib-0006] Castro, L. S. , D. Poyares , D. Leger , L. Bittencourt , and S. Tufik . 2013. “Objective Prevalence of Insomnia in the São Paulo, Brazil Epidemiologic Sleep Study.” Annals of Neurology 74, no. 4: 537–546. 23720241 10.1002/ana.23945

[jsr70255-bib-0007] Chalder, T. , G. Berelowitz , T. Pawlikowska , et al. 1993. “Development of a Fatigue Scale.” Journal of Psychosomatic Research 37, no. 2: 147–153. 8463991 10.1016/0022-3999(93)90081-p

[jsr70255-bib-0008] Cintra, F. , L. R. A. Bittencourt , R. Santos‐Silva , et al. 2012. “The Association Between the Framingham Risk Score and Sleep: A São Paulo Epidemiological Sleep Study.” Sleep Medicine 13, no. 6: 577–582. 22516609 10.1016/j.sleep.2011.12.016

[jsr70255-bib-0009] de Mello, M. T. , F. V. Narciso , S. Tufik , et al. 2012. “Sleep Disorders as a Cause of Motor Vehicle Collisions.” International Journal of Preventive Medicine 4, no. 3: 246–257. PMC3634162 23626880

[jsr70255-bib-0010] Drager, L. F. , D. V. Pachito , R. Morihisa , P. Carvalho , A. Lobao , and D. Poyares . 2022. “Sleep Quality in the Brazilian General Population: A Cross‐Sectional Study.” Sleep Epidemiology 2: 100020.

[jsr70255-bib-0011] Drager, L. F. , R. B. Santos , D. Pachito , C. S. Albertini , F. H. Sert Kuniyoshi , and A. L. Eckeli . 2024. “Inequalities in the Access to Diagnosis and Treatment of Obstructive Sleep Apnea in Brazil: A Cross‐Sectional Study.” Journal of Clinical Sleep Medicine 20, no. 5: 735–742. 38169439 10.5664/jcsm.10976 PMC11063704

[jsr70255-bib-0012] Faria, A. , A. H. Allen , N. Fox , N. Ayas , and I. Laher . 2021. “The Public Health Burden of Obstructive Sleep Apnea.” Sleep Science 14, no. 3: 257–265. 35186204 10.5935/1984-0063.20200111 PMC8848533

[jsr70255-bib-0013] Fernandes, G. L. , J. R. da Silva Vallim , V. D'Almeida , S. Tufik , and M. L. Andersen . 2024. “The Effects of Social Jetlag and Sleep Variability on Sleepiness in a Population‐Based Study: The Mediating Role of Sleep Debt.” Journal of Sleep Research 33, no. 2: e14043. 10.1111/jsr.14043. 37691450

[jsr70255-bib-0014] Fernandes, G. L. , S. Tufik , and M. L. Andersen . 2023. “Emergence of Different Dimensions of Sleepiness in a General Population Sample: An EPISONO Study.” Sleep Medicine 112: 46–52. 37806035 10.1016/j.sleep.2023.09.023

[jsr70255-bib-0015] GBD 2015 Obesity Collaborators . 2017. “Health Effects of Overweight and Obesity in 195 Countries Over 25 Years.” New England Journal of Medicine 377, no. 1: 13–27. 28604169 10.1056/NEJMoa1614362 PMC5477817

[jsr70255-bib-0016] Lévy, P. , M. Kohler , W. T. McNicholas , et al. 2015. “Obstructive Sleep Apnoea Syndrome.” Nature Reviews Disease Primers 1, no. 1: 15015. 10.1038/nrdp.2015.15 27188535

[jsr70255-bib-0017] Lopes, C. , A. M. Esteves , L. R. A. Bittencourt , S. Tufik , and M. T. Mello . 2008. “Relationship Between the Quality of Life and the Severity of Obstructive Sleep Apnea Syndrome.” Brazilian Journal of Medical and Biological Research 41, no. 10: 908–913. 18820762 10.1590/s0100-879x2008005000036

[jsr70255-bib-0018] Lucena, L. , D. N. Polesel , D. Poyares , et al. 2020. “The Association of Insomnia and Quality of Life: Sao Paulo Epidemiologic Sleep Study (EPISONO).” Sleep Health 6, no. 5: 629–635. 32335038 10.1016/j.sleh.2020.03.002

[jsr70255-bib-0019] Luciano, Y. M. , A. S. Porcacchia , S. Tufik , M. L. Andersen , and G. N. Pires . 2024. “Prevalence and Incidence of Co‐Morbid Insomnia and Sleep Apnea (Comisa) in São Paulo, Brazil.” Chest 165, no. 4: 1004–1008. 37993017 10.1016/j.chest.2023.11.024

[jsr70255-bib-0020] Malhotra, A. , C. R. Heilmann , K. K. Banerjee , J. P. Dunn , M. C. Bunck , and J. Bednarik . 2024. “Weight Reduction and the Impact on Apnea‐Hypopnea Index: A Systematic Meta‐Analysis.” Sleep Medicine 121: 26–31. 38908268 10.1016/j.sleep.2024.06.014 PMC11330732

[jsr70255-bib-0021] Malhotra, A. , J. E. Orr , and R. L. Owens . 2015. “On the Cutting Edge of Obstructive Sleep Apnoea: Where Next?” Lancet Respiratory Medicine 3, no. 5: 397–403. 25887980 10.1016/S2213-2600(15)00051-X PMC4431916

[jsr70255-bib-0022] Messineo, L. , J. P. Bakker , J. Cronin , J. Yee , and D. P. White . 2024. “Obstructive Sleep Apnea and Obesity: A Review of Epidemiology, Pathophysiology and the Effect of Weight‐Loss Treatments.” Sleep Medicine Reviews 78: 101996. 39244884 10.1016/j.smrv.2024.101996

[jsr70255-bib-0023] Netzer, N. C. , R. A. Stoohs , C. M. Netzer , K. Clark , and K. P. Strohl . 1999. “Using the Berlin Questionnaire to Identify Patients at Risk for the Sleep Apnea Syndrome.” Annals of Internal Medicine 131, no. 7: 485–491. 10.7326/0003-4819-131-7-199910050-00002. 10507956

[jsr70255-bib-0025] Sander, M. , B. Oxlund , A. Jespersen , et al. 2015. “The Challenges of Human Population Ageing.” Age and Ageing 44, no. 2: 185–187. 10.1093/ageing/afu189. 25452294 PMC4339729

[jsr70255-bib-0026] Santos‐Silva, R. , S. Tufik , S. G. Conway , J. A. Taddei , and L. R. A. Bittencourt . 2009. “Sao Paulo Epidemiologic Sleep Study: Rationale, Design, Sampling, and Procedures.” Sleep Medicine 10, no. 6: 679–685. 10.1016/j.sleep.2008.11.001. 19230759

[jsr70255-bib-0027] Togeiro, S. M. , G. Carneiro , F. F. Ribeiro Filho , et al. 2013. “Consequences of Obstructive Sleep Apnea on Metabolic Profile: A Population‐Based Survey.” Obesity 21, no. 4: 847–851. 10.1002/oby.20288. 23712988

[jsr70255-bib-0028] Tufik, S. , R. Santos‐Silva , J. A. Taddei , and L. R. A. Bittencourt . 2010. “Obstructive Sleep Apnea Syndrome in the Sao Paulo Epidemiologic Sleep Study.” Sleep Medicine 11, no. 5: 441–446. 20362502 10.1016/j.sleep.2009.10.005

[jsr70255-bib-0029] Zeng, X. , Y. Ren , K. Wu , et al. 2023. “Association Between Smoking Behavior and Obstructive Sleep Apnea: A Systematic Review and Meta‐Analysis.” Nicotine & Tobacco Research 25, no. 3: 364–371. 35922388 10.1093/ntr/ntac126 PMC9910143

