# Supplementary material for: Prevalence and Risk Factors for Obstructive Sleep Apnea in São Paulo: Findings From the 4th Edition of the EPISONO Study
Source: J Sleep Res. 2026 Jan 27;35(3):e70255. doi: 10.1111/jsr.70255 (PMC13193509; doi:10.1111/jsr.70255)
Supplement: Supplementary file 1 — Table S1: Collinearity parameters from the binary logistic regression model. [file JSR-35-e70255-s001.docx]

**Supplementary Table S1**. Collinearity parameters from the binary logistic regression model.

|  | **VIF** | **Tolerance** |
| --- | --- | --- |
| Age | 1.054 | 0.948 |
| BMI | 1.010 | 0.990 |
| Gender | 1.049 | 0.954 |
| SEC | 1.004 | 0.996 |

Abbreviations: BMI=body mass index; SEC=socio-economic classification; VIF=variance inflation factor.
